# First Steps towards the Development of Epigenetic Biomarkers in Female Cheetahs (*Acinonyx jubatus*)

**DOI:** 10.3390/life12060920

**Published:** 2022-06-20

**Authors:** Alexandra Weyrich, Tania P. Guerrero-Altamirano, Selma Yasar, Gábor Á. Czirják, Bettina Wachter, Jörns Fickel

**Affiliations:** 1 Department of Evolutionary Genetics, Leibniz Institute for Zoo and Wildlife Research, Alfred-Kowalke-Str. 17, 10315 Berlin, Germany; tania.guerrero@duke.edu (T.P.G.-A.); selmayasar94@outlook.de (S.Y.); 2 Program in Genetics and Genomics, Duke University, Durham, NC 27708, USA; 3 Institute for Biochemistry and Biology, University of Potsdam, Karl-Liebknecht-Str. 24-25, 14476 Potsdam, Germany; 4 Department of Wildlife Diseases, Leibniz Institute for Zoo and Wildlife Research, Alfred-Kowalke-Str. 17, 10315 Berlin, Germany; czirjak@izw-berlin.de; 5 Department of Evolutionary Ecology, Leibniz Institute for Zoo and Wildlife Research, Alfred-Kowalke-Str. 17, 10315 Berlin, Germany

**Keywords:** animals under human care, captivity, carnivore, DNA methylation, felidae, free-ranging, wildlife

## Abstract

Free-ranging cheetahs (*Acinonyx jubatus*) are generally healthy, whereas cheetahs under human care, such as those in zoological gardens, suffer from ill-defined infectious and degenerative pathologies. These differences are only partially explained by husbandry management programs because both groups share low genetic diversity. However, mounting evidence suggests that physiological differences between populations in different environments can be tracked down to differences in epigenetic signatures. Here, we identified differentially methylated regions (DMRs) between free-ranging cheetahs and conspecifics in zoological gardens and prospect putative links to pathways relevant to immunity, energy balance and homeostasis. Comparing epigenomic DNA methylation profiles obtained from peripheral blood mononuclear cells (PBMCs) from eight free-ranging female cheetahs from Namibia and seven female cheetahs living in zoological gardens within Europe, we identified DMRs of which 22 were hypermethylated and 23 hypomethylated. Hypermethylated regions in cheetahs under human care were located in the promoter region of a gene involved in host-pathogen interactions (*KLC1*) and in an intron of a transcription factor relevant for the development of pancreatic β-cells, liver, and kidney (*GLIS3*). The most canonical mechanism of DNA methylation in promoter regions is assumed to repress gene transcription. Taken together, this could indicate that hypermethylation at the promoter region of *KLC1* is involved in the reduced immunity in cheetahs under human care. This approach can be generalized to characterize DNA methylation profiles in larger cheetah populations under human care with a more granular longitudinal data collection, which, in the future, could be used to monitor the early onset of pathologies, and ultimately translate into the development of biomarkers with prophylactic and/or therapeutic potential.

## 1. Introduction

The cheetah is classified as vulnerable by the International Union for Conservation of Nature (IUCN) Red List of Species because it is facing several long-term survival challenges [1]. Today, free-ranging cheetahs occur in only 9% of their historical distribution range [2]. Human-carnivore conflicts, habitat loss, and fragmentation have caused marked geographical isolation and species decline [2,3]. Hence, ex-situ conservation efforts such as captive breeding programs are essential to support healthy populations for potential reintroduction programs in zoological gardens and other facilities where cheetahs are under human care [4,5]. In the past, cheetahs were often difficult to breed in zoological gardens [6,7], but more recently, breeding success has improved in some facilities due to a better understanding of cheetah reproduction physiology, improved husbandry, and better breeding management [8,9,10,11]. Cheetahs under human care are, though, still susceptible to a range of infectious and degenerative diseases, which further challenge healthy populations [12,13,14,15,16]. In contrast, studies on free-ranging cheetahs revealed that cheetahs in the wild have a high reproductive performance and are clinically healthy [11,13,17,18,19].

Previously, the impaired reproductive performance and health of cheetahs under human care were attributed to the low genetic diversity of the species [20]. The low genetic diversity has been studied in both free-ranging cheetahs and conspecifics under human care at several levels, including numbers of allozymes, single nucleotide variation, and number and diversity of alleles at microsatellite loci and at the major histocompatibility complex (MHC) [20,21,22,23]. The MHC is considered one of the most variable gene complexes in mammals and has an important function in the immune response of infected host individuals [24]. A study on the global cheetah population demonstrated that the genetic diversity of cheetahs was higher than previously described in felids [25]. Based on mitochondrial DNA and microsatellites, the genetic diversity in cheetahs was higher than in tigers *(Panthera tigris*) and pumas (*Puma concolor*) and similar to domestic cats (*Felis catus*), lions (*Panthera leo),* and pumas, respectively [25]. This increases the chances that further evidence-based improvements in husbandry management will likely be successful in improving the reproductive performance and health of cheetahs under human care [11,18,26].

In addition, there is mounting evidence that gene-environment interactions (GEIs) are strong candidates to understand physiological differences in study populations living across different environments [27,28,29,30,31,32]. One way by which such GEIs can be potentially linked to mechanisms is epigenetic modifications. Epigenetic mechanisms consist of enzymes and chemical tags that are responding to environmental cues and orchestrate stable and transient gene expression changes [33]. These changes provide the plasticity needed in embryogenesis, development, and in higher-order physiological and behavioral responses [27,34]. There are three known forms of epigenetic modifications: histone modifications, non-coding RNAs, and DNA methylation. DNA methylation is the most studied mechanism due to its stability and potential transgenerational effects [29,35,36,37]. In mammals, it occurs mainly at sites known as cytosine-phosphate-guanine dinucleotides (CpG). Methylations in promoter or enhancer regions of genes generally repress gene transcription, while demethylation leads to transcriptional activation [38]. Epigenetic modifications are vital to embryonic development, such as imprinting, cell differentiation, and transposon silencing [39]. Its alterations are associated with phenotypic diversity and a range of pathologies, including cancer and metabolic, autoimmune, and behavioral disorders [39,40]. Although methylation patterns remain relatively stable over cell divisions, they can be altered by responses to environmental stressors, which, in turn, may influence the expression of health-related traits [27].

Only a few studies have so far investigated the contribution of DNA methylation to disease susceptibility in natural systems [41]. So far, no study has focused on a comparison between free-ranging individuals and conspecifics of the same species under human care. For such a comparison, it is desirable to minimize the effect of highly diverse genotypes on phenotypic traits to reduce genetic effects. Thus, comparative epigenetic studies should be carried out in species with low genetic variability, making the cheetah an ideal study species to identify differentially methylated regions between free-ranging cheetahs and conspecifics under human care. The discovery of differentially methylated regions (DMRs) is the first step towards the detection of potential methylation biomarkers, which then need to be validated for robustness and reproducibility [42].

With the current study, we aimed to identify epigenetic patterns that help explain the observed differences in reproductive performance and disease susceptibility between free-ranging cheetahs and their conspecifics in European zoological gardens. We concentrated on females to eliminate sex effects and because we had better knowledge of the reproductive performance of free-ranging females than that of males. Our long-term aim is to make differentially methylated regions applicable as biomarkers to monitor reproductive and health status in cheetahs under human care. This will hopefully allow us to further improve husbandry conditions and the success of ex-situ conservation programs but will also be useful for population health monitoring in the wild.

## 2. Materials and Methods

### 2.1. Study Animals

Within the long-term Cheetah Research Project of the Leibniz Institute for Zoo and Wildlife Research (Leibniz-IZW, www.cheetah-research.org), free-ranging Namibian cheetahs are captured in box traps at cheetah marking trees, as previously described [19,43]. Following capture, the animals were immobilized, fitted with GPS collars, and underwent a medical check-up, during which, among other samples, blood samples were collected [19,44]. For this study, we randomly selected samples from eight free-ranging female cheetahs. Three females had offspring, two females were in estrus (determined by their reddened vulva and discharge), and the remaining three females were neither lactating (indicated by their nipples covered with fur), nor pregnant in an advanced state (no fetuses were palpated), and also not in estrus (Table 1). All females were clinically healthy.

To receive fresh blood samples from cheetah females in zoological gardens, we approached the coordinator of the European Studbook for Southern Cheetahs and asked for the distribution of a letter to zoological gardens in Germany and adjacent countries. As soon as we were informed that a cheetah was immobilized or euthanized, we organized the transport of blood to the Leibniz-IZW. We received blood samples of seven female cheetahs from zoological gardens in Germany, France, and Denmark (Table 1). The samples were from three clinically healthy adult females and four terminally ill females that had to be euthanized. Two of the latter four animals were cubs of six and seven months of age, respectively. Some females were related to each other (Table 1). We focused on peripheral blood mononuclear cells (PBMCs) because they reflect the immune system, which was the focus of our study [45], and because samples such as hair, feces, or saliva contain a low amount of endogenous DNA and bear the risk of contamination with various agents.

### 2.2. Sample Collection and PBMC Isolation

Blood samples of free-ranging females and conspecifics in zoological gardens were collected in either heparin or citrate vacutainers (Fisher Scientific, Hagen, Germany) and transported at 4 °C to the field laboratory or the Leibniz-IZW, respectively. For free-ranging females, PBMCs were isolated in a laminar flow hood within 26 h (15.7 ± 5.5 h, mean ± standard deviation (SD)) after blood collection, while for females in zoological gardens, PBMCs were isolated within 18 h (10.7 ± 6.2, mean ± SD) after blood collection. PBMCs are blood cells with a round nucleus, including lymphocytes (T cells, B cells, NK cells) and monocytes. DNA methylation studies are often aggravated by mixtures of many different cell types in tissues because different cell types exhibit differential DNA methylation at many genomic regions [30,46,47]. PBMCs reduce such aggravation. To isolate PBMCs, whole blood samples were first diluted with the same volume of 0.9% NaCl solution. Then, for each sample, three 15 mL tubes were filled with a biocoll separating solution (Biochrom GmbH, Berlin, Germany) up to a volume that corresponded to a third of the volume of the diluted NaCl-blood sample. The latter were pipetted carefully into each tube on top of the biocoll solution to maintain the density layers. The tubes were then centrifuged for 20 min at 600× *g* without deceleration to generate a density gradient. The white clouds representing the immune cells were then carefully recovered and transferred into a new 15 mL tube filled with 10 mL PBS-EDTA (pH 7.4) solution. The tubes were then centrifuged for 15 min at 400× *g*, after which the supernatant was discarded and the cell pellet carefully dissolved in 10 mL PBS-EDTA solution. The centrifugation was repeated, and the cell pellet was transferred into a 4 °C cold water bath, where it was supplemented drop by drop with a 3 mL freezing medium (90% fetal bovine serum, 10% DMSO). The newly obtained PBMC solutions were mixed via pipetting and placed in aliquots in a MrFrosty freezing container (Merck, Taufkirchen, Germany) at −80 °C. In this freezing container, samples cool down slowly at a freezing rate of −1 °C per minute. The vials from Namibia were transferred into liquid nitrogen after at least two hours in the freezing container until transportation to the Leibniz-IZW, Berlin, Germany. Transport was in full compliance with the Convention on International Trade in Endangered Species (CITES). At the Leibniz-IZW, samples were stored at −80 °C until further analysis.

All handling and sampling of the free-ranging cheetahs were performed by a veterinarian or para-veterinarian and, for the cheetahs under human care, by the respective zoo veterinarian, ensuring compliance with animal welfare regulations. All experimental procedures, including animal immobilization and sample collection in Namibia, were approved by the Internal Ethics Committee of the Leibniz Institute for Zoo and Wildlife Research (Leibniz-IZW, permit number 2002-04-01) and authorized by the Ministry of Environment, Forestry, and Tourism of Namibia (permit numbers 1514/2011, 1689/2012, 1813/2013, 1914/2014, 2067/2015).

### 2.3. DNA Extraction and Enrichment of Methylated DNA

Genomic DNA from PBMCs was extracted using the QIAamp DNA Blood Mini Kit (Qiagen, Hilden, Germany) according to the manufacturer’s instructions. For sequencing, DNA was sheared into 300–400 base pair (bp) long fragments via sonication with a Covaris SonoLab 7.1 M220. Smaller DNA fragments were removed using Ampure Beads XP (Beckmann Coulter GmbH, Krefeld, Germany). Visual control of successful DNA shearing and removal of fragments <100 bp was performed using a Tape station 2200 (Agilent Technologies, Waldbronn, Germany). Methylated DNA was captured by covalent binding to biotinylated methyl-binding-protein 2 (MBD2) and eventually bound to Streptavidin-coated Dynabeads using the MethylMiner Methylated DNA Enrichment Kit (both Thermo Fisher Scientific, Darmstadt, Germany). The meDNA-MBD2-Dynabead complex was extracted using a magnetic rack.

### 2.4. Library Preparation and High Throughput Sequencing

Illumina sequencing libraries with the methylated DNA were prepared using the NEBNext^®^ Ultra™ II DNA Library Prep Kit (Frankfurt, Germany). End-repair and adaptor ligation followed the protocol using the methylated adaptor #E7536AA. Size selection was performed with SPRI beads (AMPure Beads XP, Beckmann Coulter GmbH, Krefeld, Germany). DNA samples from cheetahs were prepared with a previously established in-house protocol that consisted of a first right-side size selection step with 0.6×, followed by left-side size selection with a ratio of 1.8× of the supernatant fraction. Samples were pooled and sequenced in two batches within six months (Table 2). Library containing tubes were protected with parafilm, packed with ice blocks, and taken to the Berlin Center for Genomics in Biodiversity Research (BeGenDiv) to be sequenced on an Illumina NextSeq platform. Due to organizational and logistical reasons, batch 1 was sequenced using the NextSeq 500/550 High Output v2 kit (paired-end sequencing PE 75 bp reads, 150 cycles) and batch 2 was sequenced using the NextSeq 500/550 High Output v2 kit (single-end sequencing SE 75 bp reads, 75 cycles, 400M reads). Thus, for batch 2, the read output was half the size of batch 1, which we corrected for within the analysis by focusing on single reads only.

### 2.5. Bioinformatics

Raw reads were bioinformatically computed to clean reads (Supplementary Table S1; Table 1). Adapters were clipped using the software cutadapt v2.4 [48], and reads were filtered for a Phred quality score of ≥33 using Trimmomatic (v.0.39). Clean reads were mapped to the cheetah reference genome Aci_jub_2 (Acinonyx jubatus, 2 https://www.ncbi.nlm.nih.gov/assembly/GCF_003709585.1, accessed on 1 August 2021, uploaded to National Center for Biotechnology Information (NCBI), U.S. National Library of Medicine in 2018) using the bowtie 2 mapper v3.5.1 [49]. Data were sorted and duplicates removed using SAMtools v1.3 [50]. Together with the reference sequence (RefSeq), an annotation file is also publicly available (https://www.ncbi.nlm.nih.gov/assembly/GCF_003709585.1, accessed on 1 August 2021) and was used for annotation. The annotation of the cheetah genome was generated by the NCBI Eukaryotic Genome Annotation Pipeline (https://www.ncbi.nlm.nih.gov/genome/annotation_euk/process/, accessed on 1 August 2021), which is an automated pipeline that annotates genes, transcripts, and proteins on draft and finished genome assemblies. The RefSeq assemblies that are annotated by NCBI are copies of the genome assemblies that are publicly available in INSDC (DDBJ, ENA, and GenBank), wherefore all can be applied for analysis.

### 2.6. Methylation Analysis

The more genomic regions are methylated in a DNA, the more MBD protein will bind and can be captured. Thus, higher methylation in a region will lead to a higher number of sequenced reads from that region. For the comparative analysis, we used a combination of two R packages, MEDIPS [51]; https://bioconductor.org/packages/release/bioc/html/MEDIPS.html, accessed on 1 August 2021) and EdgeR [52]; https://bioconductor.org/packages/release/bioc/html/edgeR.html, accessed on 1 August 2021). Several quality controls are implemented in the MEDIPS software, including (i) a coverage saturation analysis and (ii) a coverage correlation of read counts per sample. The coverage saturation helps to identify an optimal window size for tilling the genome into non-overlapping consecutive genomic regions (“windows”, ‘ws’), which is required for the read count analysis of the sequencing reads. Within the saturation analysis (i), the sequencing reads for scaffold0 are randomly split into distinct subsets of increasing size and correlations are iteratively calculated between such artificially created technical replicates and the real data [53]. The best correlation determines the best window size (in bp) for the sliding windows’ analysis, in accordance with the mean DNA fragment size used for sequencing. Best estimated correlations were determined at a window size of 200 bp. The coverage correlation (ii) among all reads (and all scaffolds) calculates pairwise correlation coefficients between each sample based on read counts per window (read depth). Samples with lower read coverage can be visually identified in the correlation matrix. Samples were similar in their average read counts (shown by high pairwise correlations).

We accounted for batch differences (paired-end (PE) vs. single-end (SE) sequencing approaches) by applying the parameters “paired = FALSE” (only read 1 was used) and “extend = 350” (extending the single-end reads up to 350 bp length) in MEDIPS (Lienhard et al., 2014). With this approach, sequencing reads were adjusted between both sequencing approaches (PE and SE) by using only read 1 of the PE sequencing and by using the single-end read of the SE sequencing and extending the single-end reads to 350 bp, which is the average DNA fragment size. In EdgeR, we accounted for a potential batch effect by including “batch” as a covariate when fitting the model (Robinson et al., 2010). As a threshold, a minimal coverage ≥ 10 reads per window among all individuals was set. Windows above this threshold were used in EdgeR, which calculates the relative read count (and thus methylation) difference per window by using a negative binomial distribution per window. Significance of differences was tested using the likelihood ratio test for each window among individuals of both groups. The resulting *p*-values were corrected for multiple testing [54] to control for false discovery rate (FDR) [53]. Neighboring windows with adjusted *p*-values ≤ 0.05 were merged into differentially methylated regions (DMRs). DMRs were then annotated to their known functional genomic region, including promoters, introns, exons, splice sites, and the untranslated regions (3′UTRs, 5′UTRs) of genes, as well as to intergenic regions using the R package ‘BS genome’ (v.1.60; https://bioconductor.org/packages/release/bioc/html/BSgenome.html, accessed on 1 August 2021), applying the annotated cheetah reference genome. The STRING online tool [55,56]; https://string-db.org, accessed on 20 January 2022; mouse genome used as reference) was applied to detect protein–protein interactions and potential pathway assignments of the KLC1 protein whose gene promoter had been methylated. 

## 3. Results

### 3.1. Sequencing Results and Differentially Methylated Regions

Mapping rates ranged between 83.66% and 98.61%, with an average mapping rate of 96.33% for sequence reads of the free-ranging female cheetahs and 96.08% for the reads of female cheetahs under human care (Table 2 and Supplementary Table S1).

### 3.2. Comparative Methylation Analysis

**Figure 1:**
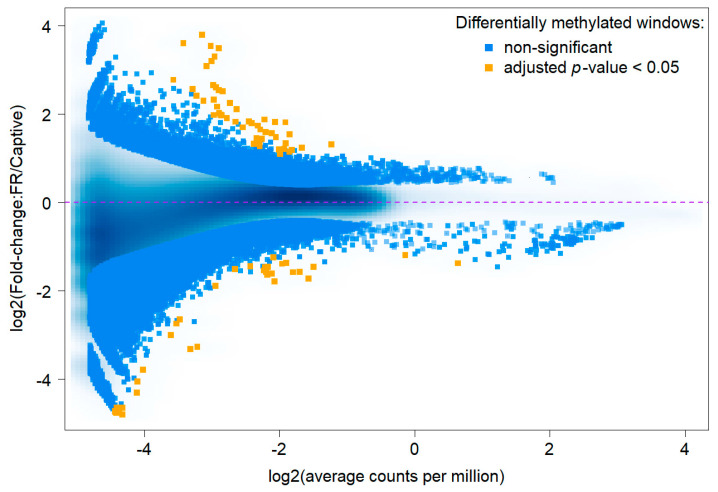
The MA-plot visualizes the log-fold change in methylated read counts per window (size 200 bp), with hypermethylation above average (>0) and hypomethylation below average (<0). Significant differentially methylated windows with adjusted *p*-value < 0.05 are highlighted in orange, non-significant in blue.

For coverage assessment, the genome was divided in silico into 11,925,794 windows of 200 bp in length. Out of these, 7,290,473 windows had a coverage ≥ 10 across all 15 samples, and as such qualified to be included for testing for differential methylation. The M (log ratio of fold change, *y*-axis) vs. A (average log read counts per million, *x*-axis) plot visualizes the methylation differences per window between measurements taken of both groups (free-ranging vs. under human care), by transforming the data onto M and A scales, and plotting these values (MA-plot). With an increase in *p*-value stringency, the number of windows reduces. We focused on regions with adjusted *p*-value ≤ 0.05 and detected 85 significantly differentially methylated windows (Figure 1, orange dots).

We obtained 45 differentially methylated regions (DMRs), of which 22 were hypermethylated and 23 were hypomethylated in free-ranging (F) females compared to conspecifics under human care (C) (Table 3). Most DMRs were overlapping with intergenic regions (Table 3). Few DMRs overlapped with either an intron within the gene body or with a promoter (Table 3), but not with a coding region, a splice site, or a 5′ or 3′UTR.

### 3.3. Genes Overlapped by DMRs and Potential Biomarkers

As a first step towards identifying functional relevant regions and potential biomarkers, we focused on the 15 DMRs that overlapped genes, i.e., introns or promoters, out of the total 45 DMRs detected in the inter-group comparison. Among the 14 DMRs overlapping introns, 8 were hypermethylated and 6 hypomethylated in female cheetahs under human care. The one promoter that was overlapped by a DMR was hypermethylated in the female cheetahs under human care (Table 4, Supplementary Table S2).

We detected DMRs overlapping genes with potential relevance for the health of cheetahs under human care (Table 4, marked in bold), the immune system (Table 4, marked with superscript a), the energy balance (Table 4, marked with superscript b), and homeostasis (Table 4, marked with superscript b, c).

### 3.4. DMRs as Potential Biomarkers

**Figure 2:**
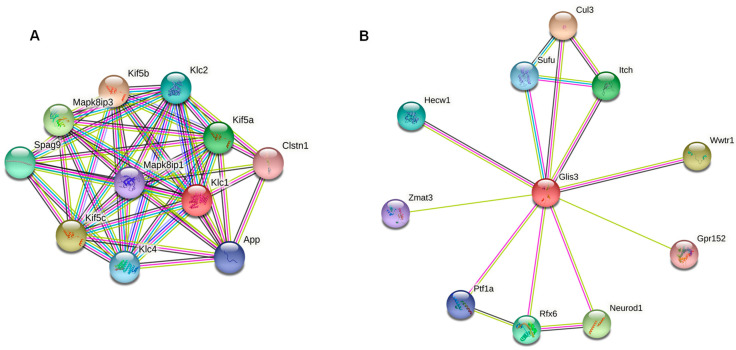
Network of interactions between (**A**) the protein *KLC1* (red circle) and other proteins of the kinesin pathway (String online tool), (**B**) the transcription factor *GLIS3* (red circle) and its downstream-regulated genes.

**Figure 3:**
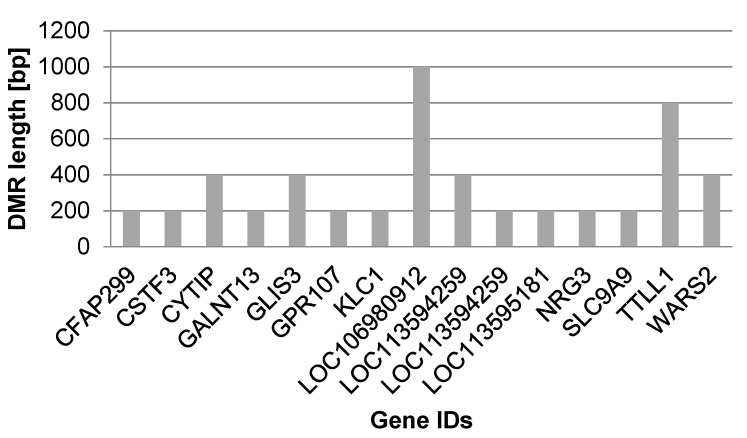
Bar chart displaying the 15 genes overlapped by differentially methylated regions (DMRs) with respect to DMR lengths in base pairs [bp].

Big datasets (obtained from -omic technologies) allow the detection of numerous DMRs. Although they are all biologically meaningful, their (regulatory) function can often only be inferred if additional information on the affected genome region is available and accessible (e.g., from annotations and/or transcriptome analyses). Thus, for the first assessment of DMRs as potential biomarkers, we suggest focusing on DMRs with known characteristics which may include: overlap with a gene of stage relevant function (Figure 2), overlap with a downstream regulation element (e.g., transcription factors; Figure 2B), or overlap with other relevant elements in the genome (e.g., enhancers, promoters), and/or the length of DMR (Figure 3, Table 2 and Table 4).

DMRs meeting these criteria will be described below, thereby focusing on three genes and intergenic regions. The first gene, coding for Kinesin light chain protein 1 (*KLC1*), was the only one with methylation changes in its promoter region; it was hypermethylated in female cheetahs under human care. The gene is important in intracellular transports and antiviral and antibacterial defenses and is part of a great network of interacting proteins (Figure 2A, red dot). The second gene, Zinc finger protein glis3 isoform (*GLIS3*), encodes a transcription factor with downstream regulatory function (forming the hub of the network, Figure 2B, red dot), which was hypermethylated in its first intron in animals under human care. The third gene with known function and overlapped by a long DMR consisting of five adjacent windows with significant differential methylation was the *TTL* Family Tubulin Polyglutamylase Complex Subunit L1 (*TTLL1*), involved in microtubule cytoskeleton organization and in a wide range of systemic functions (Figure 3, Table 4).

The two longest DMRs did not overlap genes but intergenic regions and had a length of 2000 bp which equals 10 neighboring windows (min DMR length 200 bp, max DMR length 2000 bp) (Table 5). The longest DMRs were located within the same scaffold (NW_020836466.1), which, however, lacks gene annotation. Both long DMRs were hypermethylated in female cheetahs under human care.

## 4. Discussion

In this study, we examined whether gene-environment interactions (GEIs) via DNA methylation contribute to disease susceptibility differences between free-ranging female cheetahs and conspecifics under human care. For this, we investigated the genome-wide CpG methylation differences in PBMCs in a group of free-ranging female cheetahs from Namibia and female cheetahs from different zoological gardens in Europe. Cheetahs are particularly suitable for such an epigenetic study because they have low genetic variability [20,21,22,23,25]. This minimizes potential confounding genetic effects, including those induced through relatedness between animals. Overall, methylation analysis revealed 45 differentially methylated regions (DMRs), which were equally distributed between hypomethylated (n = 23) and hypermethylated (n = 22) genomic regions.

These findings point to a function of DNA methylation in the cheetahs under human care, which may, in the future, provide biomarkers to assess their health status. Besides cheetahs, there are multiple species in which husbandry conditions have been shown to induce detrimental traits within one or just a few generation(s) that are absent in their free-ranging conspecifics [57,58,59]. Epigenetic modifications are responses to environmental stimuli, including diet, stress, exercise, sociality, and climatic conditions, regulate gene expression without altering DNA sequences, and can change within the lifetime [28,29,35,36,37].

Our results show differentially methylated regions between free-ranging female cheetahs and conspecifics under human care in genes which may be involved in disease susceptibility of the latter, as they are relevant to immune response (*KLC1*, *GLIS3*, *TTLL1*), energy balance, and homeostasis (*TTLL1*, *SLC9A9*, *WARS2*, *GLANT13*). We did not detect a differentially methylated gene that is known to directly influence female reproduction. These genes can, however, be regulated indirectly by downstream regulation of transcription factors, such as *GLIS3*, which was differentially methylated in our study.

### 4.1. Differential Methylated Genes in Cheetahs under Human Care

Promoters are the main regulatory regions of gene expression, whose methylation has been described to repress gene transcription [60]. Transcription can be repressed either directly by blocking the access of transcription factors (TFs) or indirectly by recruiting other repressive proteins with methyl-binding domains [61,62].

In our study, we detected differential methylation in the promoter of one gene, the Kinesin light chain protein 1 (*KLC1*), which was hypermethylated in female cheetahs under human care. The protein is important in intracellular transports, the mRNA surveillance pathway, and adaptive immune response. *KLC1* was shown to be active in viral, e.g., poxvirus [63,64], and bacterial infections, e.g., Salmonella [65], during the host-pathogen interactions [64]. It is documented that *KLC1* participates in *MHC-II* antigen processing and presentation via microtubule motility [64].

While the regulative function of promoter methylation has received the most attention, a study initiated in a fish species and extended across vertebrates has shown that methylation at intron 1 was inversely correlated with gene expression [66]. In this study, tissue-specific DMRs had either a positive or negative correlation with gene expression, indicative of distinct mechanisms of tissue-specific regulation. In addition, CpGs were identified in transcription factor binding motifs, which were enriched in the first intron [66]. The level of methylation tended to increase with the distance from the first exon–intron boundary, with a concomitant decrease in gene expression. As such, the DMR overlapping with the first intron in the transcription factor *GLIS3* isoform in our study might point in that direction because transcription factors regulate gene expression of many downstream genes and are thus powerful candidates for both the effect of human care on cheetahs and as a potential biomarker. The Zinc finger protein *GLIS3* isoform 1 was hypermethylated in its first intron, which may cause a repression of the same and lower activity of its downstream genes. *GLIS3* mutations have been detected in relation to neonatal, type 1, and type 2 diabetes, reflecting its function in pancreatic β-cells, where it is a drug candidate for treating a broad range of *GLIS3*-associated diabetic patients [67]. A downstream effect of *GLIS3* expression on reproduction (i.e., embryo development) cannot be excluded, as it has been reported to be involved in congenital glaucoma, hepatic fibrosis, polycystic kidneys, developmental delay, facial dysmorphism, osteopenia, sensorineural deafness, choanal atresia, craniosynostosis, and pancreatic exocrine insufficiency. Similar pathologies have been reported in cheetahs under human care [68,69], providing further support for the *GLIS3* isoform 1 as a putative biomarker.

Another protein with a reported role in the immune system, which was differentially methylated between the two groups in our study, is the *TTL* Family Tubulin Polyglutamylase Complex Subunit L1 (*TTLL1*). *TTLL1* was hypermethylated in its intron, and the DMR that *TTLL1* was part of overlapped a long region (Figure 3). The length of intronic DMRs has been proposed as a potential classification marker in cancer cells [70] and may also be of functional importance in cheetahs under human care. *TTLL1* has enzymatic activity and is a member of a large family of proteins with a *TTL* homology domain that regulates the dynamics of the microtubules by catalyzing the ligations of glutamate side chains of variable lengths on tubulins. *TTLL1* has a systemic function in neuronal function, cilia, flagella, sperm biogenesis, and motility and is expressed in monocytes. Interestingly, *TTLL1* is repressed in bovine PBMCs after vaccination against bovine tuberculosis (*bTB*), supporting a more effective immune response against tuberculosis, with a yet unclear relationship [71]. A potential downstream relation could lay in its involvement in *KLF4* glutamylation, which impedes its ubiquitination, thereby leading to somatic cell reprogramming and maintenance of pluripotency [72]. Furthermore, *TTLL1* and the tryptophanyl tRNA synthetase 2 (*WARS2*) are both ATP binding proteins, which are important in maintaining energy balance. While *TTLL1* was hypermethylated in female cheetahs under human care, *WARS2* was hypomethylated, indicating an interplay of these factors that is worth studying further.

Energy consumption and homeostasis require cellular ion transporter and metal-binding proteins. In our study, we detected polypeptide N-acetylgalactosaminyltransferase 13 (*GLANT13*) and solute carrier family 9 (sodium/hydrogen exchanger) member 9 (*SLC9A9*) to be hypermethylated in an intron, which hints at a diverse gene expression in cheetahs under human care compared with free-ranging cheetahs.

Because scaffold NW_020836466.1, which harbored the two longest DMRs found in our study (~2 kb, both hypermethylated in female cheetahs under human care), is not yet annotated, we can only speculate that additional trait-relevant epigenetic modification may be contained therein.

### 4.2. Biomarker Validation-Long-Term Aim

While the described DMRs mark differences between both cheetah groups, they are only the first steps towards biomarker development. The development of biomarkers basically requires four steps: (1) discovery, (2) assay development and analytical validation, (3) retrospective validation, and (4) prospective validation [42].

To validate the identified DMRs as biomarkers, they need to be stable and thus reproducible. To test their robustness, assays have to be developed specifically targeting these regions. This can be done, for example, by amplicon sequencing using bisulfite primer polymerase chain reactions (bisPCR), which can be performed at relatively low costs compared with the genome-wide approach used here. The developed assay can thus be further applied to a larger number of cheetahs from different zoological gardens (and free-ranging individuals as control animals). Analytical validation can be performed by using several test datasets, which are compared with the real dataset to estimate the probabilities of these DMRs occurring in animals under human care. A retrospective validation reverses this approach and attempts to map an unknown sample to the dataset, identifying its environmental condition, health, or reproductive status. In the last step, the prospective validation will statistically test the predictive power of a biomarker for its prospective phenotypic state. This can be done by a machine learning approach.

In addition to these first steps towards the development of epigenetic biomarkers, future studies would need to incorporate more samples of different age classes from free-ranging individuals and conspecifics under human care to correct for a potential age effect, because methylation patterns in some regions change with age [73]. Age is also a known confounder of reproductive performance in cheetahs [11,74]. Because epigenetic regulation differs across tissues [30,46,47], future studies can include different tissues (e.g., hair roots, intestinal mucosal cells from feces, or mouth mucosal cells from saliva). Ideally, samples will be accessible for non-invasive collection or from sampling during immobilization of animals and be composed of just a single cell type or at least of very few cell types [75].

## 5. Conclusions

We aimed to evaluate DNA methylation patterns as a molecular marker system that displayed differences in cheetahs under free-ranging conditions and under human care. We hope that this knowledge will help in the future to assist in and improve cheetah breeding programs, reintroductions, and homeostasis under human care conditions. Thus, we aimed to take a first step towards elucidating the triggers that lead to the trait changes seen in animals under human care conditions. DMRs can be further applied as biomarkers aiding monitoring and husbandry conditions for cheetahs and other animals in human care.

## 6. Outlook

Studies on wildlife epigenetics have addressed questions regarding factors such as changing temperatures [29], behavior [31,76,77,78], and means of coping with resource accessibility [28,32]. Here, we demonstrate that the relationship between environmental factors and the methylome can be applied to understanding mechanisms of health and disease in wildlife [79].

We believe that our results can pave the way for a health-oriented epigenetics approach and its translation into practice, both in free-ranging populations and animals under human care. DNA methylation biomarkers will become important to monitor wild populations in terms of age [80,81], health status, or effects of human disturbances. Well-characterized epigenetic patterns (i.e., epigenetic biomarkers) can be used for targeted management practices and have potential as diagnostic tools, as well as therapeutic and immunization targets in conservation strategies of endangered species.

## Figures and Tables

**Table 1:** Age, health status, and reproductive status at sampling date and relatedness among free-ranging (F) female cheetahs and conspecifics under human care (C) in zoological gardens. The age of the free-ranging females in Namibia was estimated following Caro [17], the age of the females under human care was provided by the facilities.

Animal_ID	Origin	Age	Clinical Status	Reproductive State	Relatedness	Date of Sampling (dd.mm.yyyy)	Batch
F_1	Namibia	3.5–7.0 y	clinically healthy	lactating mother with 4 cubs (21 days old)	unknown	28.04.2016	1
F_2	Namibia	3.5–7.0 y	clinically healthy	in oestrus	unknown	11.05.2013	1
F_3	Namibia	3.5–7.0 y	clinically healthy	mother with 3 cubs (5–6 months old)	unknown	09.06.2012	1
F_4	Namibia	3.5–7.0 y	clinically healthy	not breeding	unknown	14.10.2011	2
F_5	Namibia	2.0–3.5 y	clinically healthy	mother with 3 cubs (8–9 months old)	unknown	24.07.2012	1
F_6	Namibia	2.0–3.5 y	clinically healthy	in oestrus	unknown	16.10.2014	1
F_7	Namibia	2.0–3.5 y	clinically healthy	not breeding	unknown	13.12.2011	2
F_8	Namibia	13–23 m	clinically healthy	not breeding	unknown	16.04.2014	2
C_1	EEP, Zoo A	11 y	clinically healthy	not breeding	aunt of C_4 and C_5, great-aunt of C_7	23.04.2019	1
C_2	EEP, Zoo B	10 y 1 m	pendulous abdomen and emesis	not breeding, but bred successfully previously	mother of C_6	01.08.2019	2
C_3	EEP, Zoo C	9 y 9 m	arthrosis, euthanized	not breeding, but bred successfully previously	unrelated up to 3rd degree to the other females	24.09.2019	2
C_4	EEP, Zoo C	7 y 4 m	chronic kidney disease, euthanized	not breeding	niece of C_1 and cousin of C_5	24.09.2019	2
C_5	EEP, Zoo D	3 y 9 m	clinically healthy	in oestrus	niece of C_1, aunt of C_7 and cousin of C_4	10.12.2018	1
C_6	EEP, Zoo B	7 m	degenerated cerebrum, euthanized	immature	daughter of C_2	19.12.2018	1
C_7	EEP, Zoo E	6 m	severe ataxia, euthanized	immature	niece of C_5, great-niece of C_1	11.07.2018	1

“Clinically healthy” in free-ranging animals refers to an assessment after inspection by trained veterinarians and biologists in the field. “Clinically healthy” and “symptomatic” in animals in zoological gardens refers to an assessment after clinical inspection, lab tests, and/or necropsy reports from corresponding institutional medical bodies. Batch 1 was sequenced with the NextSeq 500/550 High Output v2 kit (paired-end sequencing, PE 75), whereas batch 2 was sequenced with the NextSeq 500/550 High Output v2 kit (single-end sequencing, SE 75). EEP: European Endangered Species Programs.

**Table 2:** Average sequencing results for free-ranging female cheetahs and conspecifics under human care.

Female Groups	N	Average Raw Single End Reads	Read Length [bp]	Average %GC	Clean Single End Reads	Average Mapping Rate [%]
Free-ranging (F)	8	31,435,731	20–76	52.5	30,972,291	96.33
Human care (C)	7	41,310,770	20–76	52.7	40,927,482	96.08

**Table 3:** Regions that were differentially methylated between free-ranging female cheetahs (F) and conspecifics under human care (C), and their genomic locations.

	Number of Differentially Methylated		DMRs Overlapping with Annotated Genomic Regions		
F vs. C	Windows	Regions (DMRs)	Intergenic	Introns	Promoters
Hypermethylated	50	22	13	8	1
Hypomethylated	35	23	17	6	0

Hypermethylated: regions are hypermethylated in free-ranging cheetahs compared with conspecifics under human care. Hypomethylated: regions are hypomethylated in free-ranging cheetahs compared with conspecifics under human care. DMR: differentially methylated region. Numbers in the right three columns represent numbers of DMRs overlapping these annotated genomic elements.

**Table 4:** Annotated genes overlapped by DMRs, including their annotations and length in base pairs.

Gene ID	Gene Name	Direction of Methylation in Captive Animals	Annotation	DMR Length [bp]
*CFAP299*	Cilia and flagella associated protein 299	hypo	intron	200
*CSTF3*	Cleavage stimulation factor, 3′ pre-RNA, subunit 3, 77 kDa	hyper	intron	200
*CYTIP*	Cytohesin-interacting protein isoform ×1; Cytohesin 1 interacting protein	hyper	intron	400
***GALNT13*** ^**c**^	Polypeptide n-acetylgalactosaminyltransferase 13 isoform ×3; Polypeptide N-acetylgalactosaminyltransferase 13	hyper	intron	200
***GLIS3 *** ^**a**^	Zinc finger protein glis3 isoform ×1; Uncharacterized protein; GLIS family zinc finger 3	hyper	intron	400
*GPR107*	G protein-coupled receptor 107	hypo	intron	200
***KLC1 *** ^**a**^	Kinesin light chain 1 isoform ×1; Uncharacterized protein; Kinesin light chain 1	hyper	promoter	200
*LOC106980912*	Zinc finger protein 708-like isoform ×1	hyper	intron	1000
*LOC113594259*	uncharacterized LOC113594259	hypo	intron	400
*LOC113594259*	uncharacterized LOC113594259	hypo	intron	200
*LOC113595181*	uncharacterized LOC113595181, ncRNA	hyper	intron	200
*NRG3*	Pro-neuregulin-3, membrane-bound isoform isoform ×5; Uncharacterized protein; Neuregulin 3	hypo	intron	200
***SLC9A9*** ^**c**^	Solute carrier family 9 (sodium/hydrogen exchanger), member 9; Belongs to the monovalent cation:proton antiporter 1 (CPA1) transporter (TC 2.A.36) family	hyper	intron	200
***TTLL1 *** ^**a,b**^	Low quality protein: probable tubulin polyglutamylase ttll1; Tubulin tyrosine ligase-like family, member 1	hyper	intron ^1^	800
***WARS2*** ^**b**^	Tryptophanyl tRNA synthetase 2, mitochondrial; Belongs to the class-I aminoacyl-tRNA synthetase family	hypo	intron	400

Genes in bold are of functional interest in ^a^: immune function, ^b^: energy balance, ^c^: homeostasis (ion transporter). Gene ontologies (GOterms) are listed in Supplementary Table S2. ^1^: DMR overlapping first intron. Genes in bold and underlined have additional biological relevant criteria.

**Table 5:** Intergenic regions overlapped by differentially methylated regions (DMRs), including their length.

Intergenic DMR	Number of DMRs	Mean DMR Length	SD DMR Length	Minimum Length	Maximum Length
Hypermethylated	13	492.31	499.94	200.00	2000.00
Hypomethylated	17	282.35	138.20	200.00	600.00

## Data Availability

The code is made available on https://github.com/WildlifeEpigeneticsWeyrichLab/Development-of-Epigenetic-Biomarkers-in-Female-Cheetahs.git. Reference genome is available on https://www.ncbi.nlm.nih.gov/assembly/GCF_003709585.1.

## Supplementary Materials

The following supporting information can be downloaded at: https://www.mdpi.com/article/10.3390/life12060920/s1. Table S1: Individual sequencing results for PBMC-derived and MBD-captured methyl-DNA from free-ranging females and under human care. Table S2: Annotated genes overlapped by DMRs, including their Gene Ontology (GO) terms.
