# Supplementary material for: First Steps towards the Development of Epigenetic Biomarkers in Female Cheetahs (Acinonyx jubatus)
Source: Life (Basel). 2022 Jun 20;12(6):920. doi: 10.3390/life12060920 (PMC9225391; doi:10.3390/life12060920)
Supplement: Supplementary file 1 [file life-12-00920-s001.zip › life-1715624-supplementary.pdf]

**Supplementary material**

**Supplementary Table S1:** Individual sequencing results for PBMC-derived and MBD-captured methyl-DNA from free-ranging females and under human care.

| Animal_Id | Status | Batch | Raw single end reads | %GC | Clean single end reads | Mapped reads | Mapping efficiency [%] |
|---|---|---|---|---|---|---|---|
| F_1 | Free-ranging | 1 | 22889851 | 52 | 22372654 | 22372654 | 83.66 |
| F_2 | Free-ranging | 1 | 26349606 | 54 | 26216984 | 26216984 | 96,08 |
| F_3 | Free-ranging | 1 | 32213939 | 48 | 32103436 | 32103436 | 95,4 |
| F_4 | Free-ranging | 1 | 20895459 | 47 | 20767583 | 20767583 | 95,95 |
| F_5 | Free-ranging | 1 | 25034844 | 56 | 24631774 | 24631774 | 96,78 |
| F_6 | Free-ranging | 2 | 40410591 | 55 | 39629442 | 39629442 | 98,61 |
| F_7 | Free-ranging | 2 | 44418720 | 54 | 43438663 | 43438663 | 98,48 |
| F_8 | Free-ranging | 2 | 39272835 | 55 | 38211109 | 38211109 | 93,04 |
| C_1 | Human care | 1 | 27695035 | 52 | 27594177 | 27594177 | 95,8 |
| C_2 | Human care | 1 | 32228340 | 52 | 32109179 | 32109179 | 96,85 |
| C_3 | Human care | 1 | 31035479 | 52 | 30948725 | 30948725 | 94,73 |
| C_4 | Human care | 1 | 25929318 | 52 | 25866751 | 25866751 | 96,14 |
| C_5 | Human care | 2 | 69587963 | 53 | 69458113 | 69458113 | 95,41 |
| C_6 | Human care | 2 | 40415509 | 54 | 39625842 | 39625842 | 95,95 |
| C_7 | Human care | 2 | 62283749 | 54 | 60889584 | 60889584 | 97,7 |

## Supplementary Table S2: Annotated genes overlapped by DMRs, including their Gene Ontology (GO) terms

| Gene ID | Gene name (info STRING) | GOterm_names | GOterm_ID |
|---|---|---|---|
| *CFAP299* | Cilia and flagella associated protein 299 | nucleus, cytoplasm, protein binding | GO:0005634, GO:0005737, GO:0005515 |
| *CSTF3* | Cleavage stimulation factor, 3' pre-RNA, subunit 3, 77kDa | nucleus, protein binding, RNA processing, mRNA processing, RNA binding, nucleoplasm, mRNA binding, mRNA cleavage, mRNA polyadenylation, RNA 3'-endprocessing | GO:0005634, GO:0005515, GO:0006396, GO:0006397, GO:0003723, GO:0005654, GO:0003729, GO:0006378, GO:0006379, GO:0031123 |
| *CYTIP* | Cytohesin-interacting protein isoform x1; Cytohesin 1 interacting protein | regulation of cell adhesion, cytoplasm, nucleoplasm, cytosol, cell cortex | GO:0030155, GO:0005737, GO:0005654, GO:0005829, GO:0005938 |
| ***GALNT13***[c] | Polypeptide n-acetylgalactosaminyltransferase 13 isoform x3; Polypeptide N-acetylgalactosaminyltransferase 13 | Golgi apparatus, carbohydrate binding, membrane, integral component of membrane, transferase activity, glycosyltransferase activity, metal ion binding, Golgi apparatus, polypeptide N-acetylgalactosaminyltransferase activity, O-glycan processing, Golgi membrane, polypeptide N-acetylgalactosaminyltransferase activity, protein O-linked glycosylation, polypeptide N-acetylgalactosaminyltransferase activity, Golgi membrane, protein glycosylation, protein O-linked glycosylation via threonine, protein O-linked glycosylation via serine, polypeptide N-acetylgalactosaminyltransferase activity | GO:0005794, GO:0030246, GO:0016020, GO:0016021, GO:0016740, GO:0016757, GO:0046872 , GO:0005794, GO:0004653, GO:0016266, GO:0000139, GO:0004653, GO:0006493, GO:0004653, GO:0000139, GO:0006486, GO:0018243 , GO:0018242, GO:0004653 |
| ***GLIS3***[a] | Zinc finger protein glis3 isoform x1; Uncharacterized protein; GLIS family zinc finger 3 | nucleus, regulation of transcription by RNA polymerase II, RNA polymerase II transcription regulatory region sequence-specific DNA binding, metal ion binding, DNA binding, negative regulation of transcription by competitive promoter, DNA-binding transcription factor activity, RNA polymerase II-specific, RNA polymerase II cis-regulatory region sequence-specific DNA binding, transcription by RNA polymerase II, DNA-binding transcription repressor activity, RNA polymerase II-specific | GO:0005634, GO:0006357, GO:0000977, GO:0046872, GO:0003677, GO:0043565, GO:0045944, GO:0005515, GO:0000122, GO:0000981, GO:0000978, GO:0006366, GO:0001228, GO:0001227 |
| *GPR107* | G protein-coupled receptor 107 | integral component of membrane, membrane, Golgi apparatus, nucleoplasm, plasma membrane, clathrin-coated vesicle, clathrin-dependent endocytosis, clathrin heavy chain binding, early endosome | GO:0016021, GO:0016020, GO:0005794, GO:0005654, GO:0005886, GO:0030136, GO:0072583, GO:0032050, GO:0005769 |
| ***KLC1***[a] | Kinesin light chain 1 isoform x1; Uncharacterized protein; Kinesin light chain 1 | protein binding, kinesin complex, cytoplasm, membrane, cytoskeleton, cytoplasmic vesicle, cell projection, microtubule, growth cone, cytosol, motor activity, stress granule disassembly | GO:0005515, GO:0005871, GO:0005737, GO:0016020, GO:0005856, GO:0031410, GO:0042995, GO:0005874, GO:0030426, GO:0005829, GO:0003774, GO:0035617 |
| *LOC106980912* | Zinc finger protein 708-like isoform X1 | regulation of transcription, DNA-templated, metal ion binding | GO:0006355, GO:0046872 |
| *LOC113594259* | uncharacterized LOC113594259 | NA | NA |
| *LOC113594259* | uncharacterized LOC113594259 | NA | NA |
| *LOC113595181* | uncharacterized LOC113595181 | NA | NA |
| *NRG3* | Pro-neuregulin-3, membrane-bound isoform isoform X5; Uncharacterized protein; Neuregulin 3 | nervous system development, membrane, integral component of membrane, signaling receptor binding | GO:0007399, GO:0016020, GO:0016021, GO:0005102 |
| ***SLC9A9***[c] | Solute carrier family 9 (sodium/hydrogen exchanger), member 9; Belongs to the monovalent cation:proton antiporter 1 (CPA1) transporter (TC 2.A.36) family | proton antiporter activity, proton antiporter activity, integral component of membrane, regulation of pH, sodium ion transport, cation transport, transmembrane transport, antiporter activity, membrane, integral component of membrane, sodium ion transport, ion transport, endosome, protein binding, regulation of intracellular pH, potassium ion transmembrane transport, sodium ion import across plasma membrane | GO:0015299, GO:0015385, GO:0016021, GO:0006885, GO:0006814, GO:0006812, GO:0055085, GO:0015297, GO:0016020, GO:0016021, GO:0006814, GO:0006811, GO:0005768, GO:0005515, GO:0051453, GO:0071805, GO:0098719 |
| ***TTLL1***[a,b] | Low quality protein: probable tubulin polyglutamylase ttll1; Tubulin tyrosine ligase-like family, member 1 | cytoplasm, nucleotide binding, ATP binding, cytoskeleton, cellular protein modification process, microtubule, ligase activity, protein polyglutamylation, extracellular region, microtubule cytoskeleton organization, cilium, tubulin binding, tubulin-glutamic acid ligase activity, immune response in nasopharyngeal-associated lymphoid tissue, epithelial cilium movement involved, sperm axoneme assembly, cerebellar Purkinje cell differentiation, flagellated sperm motility, axoneme assembly, mucociliary clearance, regulation of blastocyst development, ciliary basal body | GO:0005737, GO:0000166, GO:0005524, GO:0005856, GO:0006464, GO:0005874, GO:0016874, GO:0018095, GO:0005576, GO:0000226, GO:0005929, GO:0015631, GO:0070740, GO:0002395, GO:0003351, GO:0007288, GO:0021702, GO:0030317, GO:0035082, GO:0120197, GO:0120222, GO:0036064 |
| ***WARS2***[b] | Tryptophanyl tRNA synthetase 2, mitochondrial; Belongs to the class-I aminoacyl-tRNA synthetase family | ATP binding, nucleotide binding, aminoacyl-tRNA ligase activity, tRNA aminoacylation for protein translation, tryptophan-tRNA ligase activity, tryptophanyl-tRNA aminoacylation, translation, nucleoplasm, plasma membrane, mitochondrion, mitochondrial matrix, ligase activity, mitochondrial tryptophanyl-tRNA aminoacylation, vasculogenesis | GO:0005524, GO:0000166, GO:0004812, GO:0006418, GO:0004830, GO:0006436, GO:0006412, GO:0005654, GO:0005886, GO:0005739, GO:0005759, GO:0016874, GO:0070183, GO:0001570 |

Genes in bold are of functional interest in a: immune function, b: energy balance, c: homeostasis (ion transporter). Gene ontologies (GOterms) are listed in supplementary table S2. 1: DMR overlapping intron 1. Genes in bold and underlined have additional biological relevant criteria.
